# Extended Erythropoietin Treatment Prevents Chronic Executive Functional and Microstructural Deficits Following Early Severe Traumatic Brain Injury in Rats

**DOI:** 10.3389/fneur.2018.00451

**Published:** 2018-06-19

**Authors:** Shenandoah Robinson, Jesse L. Winer, Lindsay A. S. Chan, Akosua Y. Oppong, Tracylyn R. Yellowhair, Jessie R. Maxwell, Nicholas Andrews, Yirong Yang, Laurel O. Sillerud, William P. Meehan, Rebekah Mannix, Jonathan L. Brigman, Lauren L. Jantzie

**Affiliations:** ^1^Neurosurgery, Boston Children's Hospital, Harvard Medical School, Boston, MA, United States; ^2^Neurology, Boston Children's Hospital, Harvard Medical School, Boston, MA, United States; ^3^F.M. Kirby Center for Neurobiology, Boston Children's Hospital, Harvard Medical School, Boston, MA, United States; ^4^Department of Pediatrics, University of New Mexico, Albuquerque, NM, United States; ^5^Department of Pharmaceutical Sciences, University of New Mexico, Albuquerque, NM, United States; ^6^Department of Neurology, University of New Mexico, Albuquerque, NM, United States; ^7^Sports Medicine, Boston Children's Hospital, Harvard Medical School, Boston, MA, United States; ^8^Emergency Medicine, Boston Children's Hospital, Harvard Medical School, Boston, MA, United States; ^9^Department of Neurosciences, University of New Mexico, Albuquerque, NM, United States

**Keywords:** controlled cortical impact, diffusion tensor imaging, diffusivity, infant, touchscreen, cognition, cognitive flexibility

## Abstract

Survivors of infant traumatic brain injury (TBI) are prone to chronic neurological deficits that impose lifelong individual and societal burdens. Translation of novel interventions to clinical trials is hampered in part by the lack of truly representative preclinical tests of cognition and corresponding biomarkers of functional outcomes. To address this gap, the ability of a high-dose, extended, post-injury regimen of erythropoietin (EPO, 3000U/kg/dose × 6d) to prevent chronic cognitive and imaging deficits was tested in a postnatal day 12 (P12) controlled-cortical impact (CCI) model in rats, using touchscreen operant chambers and regional analysis of diffusion tensor imaging (DTI). Results indicate that EPO prevents functional injury and MRI injury after infant TBI. Specifically, subacute DTI at P30 revealed widespread microstructural damage that is prevented by EPO. Assessment of visual discrimination on a touchscreen operant chamber platform demonstrated that all groups can perform visual discrimination. However, CCI rats treated with vehicle failed to pass reversal learning, and perseverated, in contrast to sham and CCI-EPO rats. Chronic DTI at P90 showed EPO treatment prevented contralateral white matter and ipsilateral lateral prefrontal cortex damage. This DTI improvement correlated with cognitive performance. Taken together, extended EPO treatment restores executive function and prevents microstructural brain abnormalities in adult rats with cognitive deficits in a translational preclinical model of infant TBI. Sophisticated testing with touchscreen operant chambers and regional DTI analyses may expedite translation and effective yield of interventions from preclinical studies to clinical trials. Collectively, these data support the use of EPO in clinical trials for human infants with TBI.

## Introduction

Traumatic brain injury (TBI) is the leading cause of mortality and morbidity for full term infants who are born healthy (1, 2). Pediatric TBI exacerbates social and economic burden throughout the lifespan, and pediatric inpatients accrue an estimated >$1 billion in total charges for TBI-associated hospitalizations (2, 3). Young children (0–4 years) have the highest rates of TBI of any pediatric age group, though the end result of severe TBI only fully manifests as the central nervous system (CNS) fails to mature with an appropriate developmental trajectory (1, 3, 4). Indeed, children who survive early TBI are at risk for numerous chronic neurological deficits, including impairments in cognition and executive function (3, 5).

Despite the burden of severe chronic sequelae after infant TBI, no treatments are available to enhance the repair of the injured developing brain, beyond supportive therapy offered with typical critical care. A potential emerging intervention for infant TBI is erythropoietin (EPO) (6–9). EPO and its receptor EPOR, have important roles in the nervous system, independent of its hematopoietic actions (10–16). In healthy humans and rodents, recombinant EPO improves cognition and increases hippocampal long-term potentiation (17, 18). EPO is effective after numerous types of insults in the adult CNS (19–21) including TBI (22, 23). Prior data indicate that EPO crosses the blood-brain barrier via non-receptor mediated transport in both humans and rodents (10, 24). After perinatal brain injury, neural cell EPOR expression increases without concomitant EPO ligand expression (6, 7, 25–28), suggesting that exogenous EPO is potentially more effective in the developing CNS. Notably, without ligand present, unbound EPOR triggers neural cell death and exogenous EPO restores balanced EPOR signaling supporting neural cell development (12, 13, 27, 28). Previously, we have reported that extended EPO treatment after infant TBI on postnatal day 12 (P12) in rats, facilitates widespread repair of both gray and white matter, with concomitant prevention of motor deficits (7), similar to reports by other labs demonstrating that EPO improves recognition memory, hippocampal volume, and reduces cell death following TBI in a model of older pediatric TBI on P17 (8, 9).

Translation of emerging neuroreparative agents has been challenging following pediatric TBI, in part due to limited investigation using sophisticated preclinical platforms and outcome measures capable of detecting executive function and chronic diffusion tensor imaging (DTI) abnormalities (1, 29, 30). Further, a lack of sensitive, quantitative outcome measures has been implicated in the failure to detect meaningful differences in clinical improvement in TBI clinical trials (31–33). As neurodevelopmental tests are typically designed to compare age-equivalent groups of infants, and infants suffer TBI at various ages, current neurodevelopmental scales have been deemed inadequate to capture subacute (30 day) and chronic (6 month) outcomes for early phase trials in infant TBI. Thus, sensitive, reliable and reproducible, quantitative imaging measures of damage and recovery can potentially fill this void, and act as a surrogate biomarker for injury and repair. Accordingly, to fill these gaps in knowledge and more rigorously test the efficacy of potential therapeutic strategies for infants with TBI prior to translation to clinical trials, we tested the hypothesis that a touchscreen platform, analogous to the Cambridge Neuropsychological Test Automated Battery (CANTAB) in humans, could detect sophisticated differences in cognition in rats following early TBI and neurorepair with extended EPO treatment. Taken together, our data demonstrate for the first time the feasibility of sophisticated touchscreen testing of pillars of cognition in a preclinical model of severe infantile TBI. Moreover, we report that extended EPO treatment prevents cognitive and executive function deficits, and concomitant chronic and correlative DTI abnormalities in adult rats following infant TBI.

## Methods

**Figure 1:**
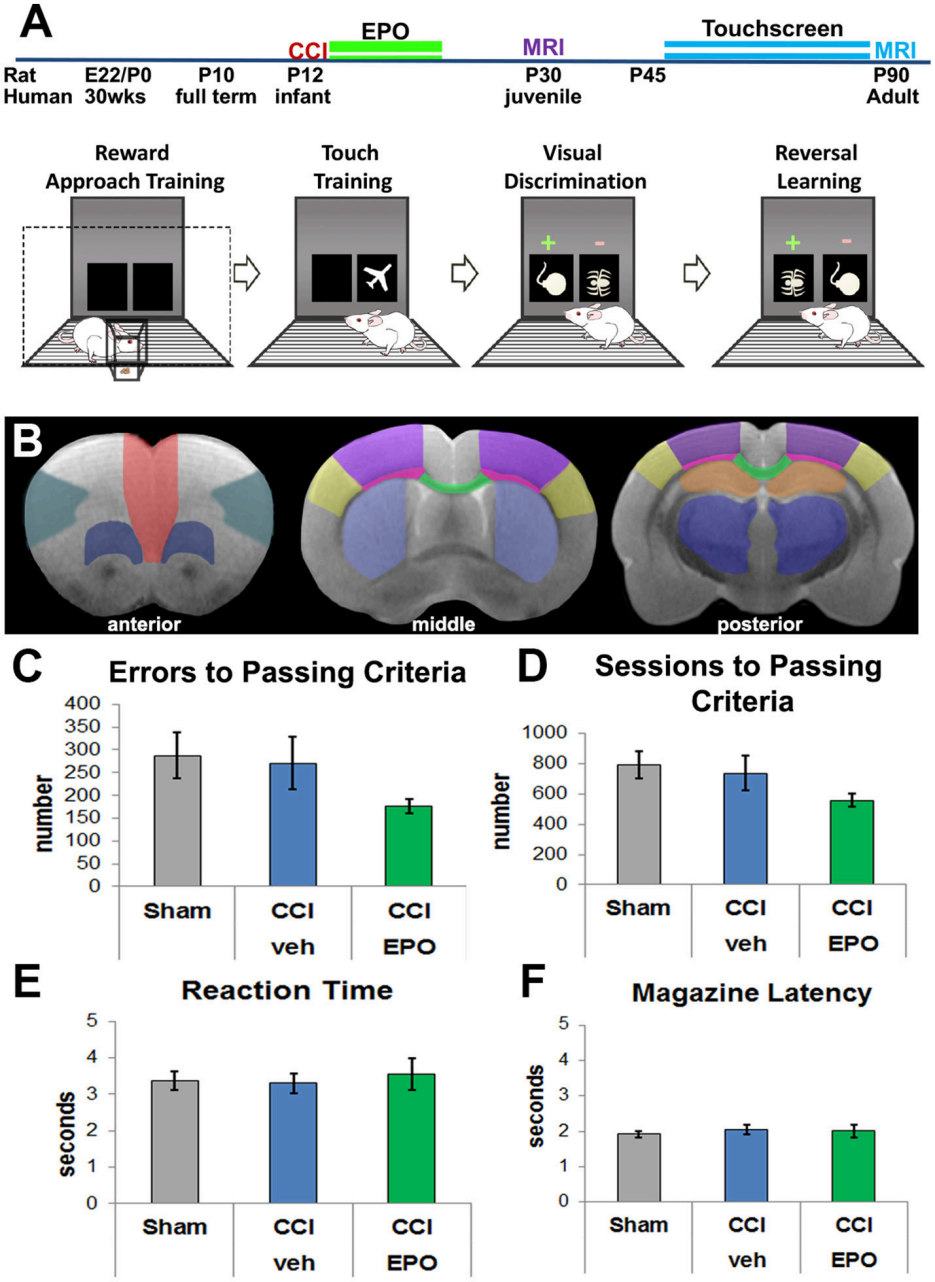
Experimental details and visual discrimination. **(A)** Experimental paradigm including pictorial summary of touchscreen training, visual discrimination, and reversal learning. Rats are first habituated to the food rewards and the touchscreen itself, and then progress to assessment of visual discrimination and cognitive flexibility. Arrows indicate passing of training and testing stages based on *a priori* criteria. The two MRI cohorts are represented by different colors. **(B)** Regions of interest for DTI analyses *anterior* (corresponding to Bregma 3.72 mm): red—medial prefrontal; teal—lateral prefrontal; dark blue—ventral prefrontal; middle (Bregma −0.12 mm) and posterior (Bregma −3.00 mm): yellow—barrel cortex, purple—lesion cortex, neon pink—lateral white matter, green—corpus callosum, light blue—striatum, orange—hippocampus, dark blue—thalamus. **(C)** All 3 groups committed a similar number of errors prior to passing visual discrimination (VD), demonstrating that injured rats can complete the VD task (*n* = 6–8). **(D)** Likewise, all 3 groups required a similar number of sessions to performance criteria. **(E)** Rats in all 3 groups displayed a similar reaction time to respond to touchscreen stimuli. **(F)** Rats also demonstrated a similar motivation to retrieve the reward for a correct response.

All procedures were performed in accordance with NIH Guide for the Care and Use of Laboratory Animals and were approved by Institutional Animal Care and Use Committees at the Boston Children's Hospital and the University of New Mexico Health Sciences Center. For each experiment, balanced numbers of male and female pups were used, and data represent true n (individual pups) from at least 2 different dams. All investigators were blinded to injury and treatment group during the conduct and analyses of each experiment. A power analysis was performed to estimate required sample size (G^*^Power 3.1.9.3) using published and preliminary data to define expected means and standard deviations for each group (7). We determined the number of samples needed for 80% power in a two-way design to establish the effect of EPO treatment. In order to detect as 20% change with 20% error, an α of 0.05, the number of animals required was 6. Separate cohorts of rats were used for imaging studies at 30 days, and touchscreen plus imaging at 90 day evaluations. For the DTI, primary outcomes were fractional anisotropy, mean diffusivity, axial diffusivity and radial diffusivity in anatomically defined regions of interest. For touchscreen analyses, the primary outcome measures were the number of errors to reach passing criteria, and number of sessions to reach passing criteria in visual discrimination. Secondary outcomes were reaction time and magazine latency. For reversal learning, the primary outcomes were percent passing, and number of correction trials. Secondary outcomes were sessions to passing criteria, errors to passing criteria, correction trials during perseveration, correction trials during learning, reaction time and magazine latency. Visual representation of our experimental design, including the progression through touchscreen stages is provided in Figure 1A.

### Controlled cortical impact

Controlled cortical impact (CCI) was delivered to Sprague-Dawley rat pups on P12 (7). Briefly, rat pups were anesthetized with isoflurane and a 5 mm diameter left craniectomy was performed. Heads were fixed in the prone position, and an air-powered piston (3 mm diameter, Amscien, Richmond, VA) delivered a CCI of 0.6 mm depth, at 6 m/s to the left parietal lobe. Pups were reared with their dams until P21, then weaned and housed in single sex pairs. Housing room lights were on from 7 a.m. to 7 p.m., with food and water available *ad libitum*. Temperature was maintained at 21 ± 1°C and humidity at 55 ± 5%.

### Erythropoietin (EPO) administration

Injured rats were randomized 24 h following CCI to receive either EPO (3000 U/kg/day, R&D Systems, Minneapolis, MN) or vehicle (sterile saline) (7). EPO or vehicle was injected intraperitoneally once daily on days 1, 2, 3, 4, 6, and 8 following CCI.

### Touchscreen testing: pretraining

Visual discrimination (VD) and reversal learning were assessed consistent with prior reports, with minor modifications for rats (34–37). Briefly, operant behavior was conducted in a sound and light attenuating chamber (Med Associates, St. Albans, VT), with a pellet dispenser and a touch-sensitive screen (Conclusive Solutions, Sawbridgeworth UK). Stimulus presentation in the response window and touches were controlled and recorded by KLimbic Software (Conclusive Solutions, Sawbridgeworth UK).

On P28, rats were reduced to and maintained at 90% free-feeding body weight. Prior to training, rats were acclimated to 40 mg food pellet reward by provision of 25 pellets/rat in a home cage. Rats were then habituated to the operant chamber and to eating from the pellet magazine. Rats retrieving at least 48 of 60 pellets in 60 min were moved to a 4-stage training regimen. Beginning on P35, rats first performed autoshaping, followed by VD training 1, 2, and 3 (36).

### Touchscreen testing: discrimination and reversal learning

Following pretraining, all rats were tested on a pairwise visual discrimination-reversal paradigm. Each rat performed daily sessions for a maximum of 60 min. For discriminative learning, two novel equiluminescent stimuli were presented in a spatially pseudo-randomized manner over 60-trial sessions (5 s inter-trial interval). Responses at one stimulus yielded a reward, whereas responses at the other stimulus resulted in 5 s time out (signaled by extinguishing the house light). Designation of the initial reward stimulus was randomized across treatment. Stimuli remained on screen until a response was made. Rats were trained *a priori* to a criterion of greater than ≥80% correct responses for two consecutive days.

Assessment of reversal learning began after VD performance criteria were attained. For this test, the designation of stimuli as correct vs. incorrect was reversed for each rat. Like VD, rats were tested on daily 60-trial sessions for reversal to an *a priori* criterion of ≥80% correct responses for two consecutive sessions. Correction trials following errors were presented, with the same stimuli, in the same spatial orientation, until a correct response was made, or the session ended. Failing criteria were set *a priori* at 24 sessions (days) for both VD and reversal.

We recorded the following dependent measures during VD and reversal: total sessions, correct responses made, errors (incorrect responses), correction errors (correction trials, reversal only), reaction time (time from touchscreen stimuli presentation to touchscreen response) and magazine latency (time from touchscreen response to reward retrieval). Discrimination performance was analyzed across all sessions required to reach criterion. To examine distinct phases of reversal (early perseverative and late learning), we analyzed errors and correction errors for sessions where performance was < 50% and from 50% to criterion, respectively (36–39).

### Magnetic resonance imaging (MRI)

At P30 (1 month of age) or P90 (3 months of age), rats were anesthetized and perfused with phosphate-buffered saline, followed by 4% paraformaldehyde. Brains were post-fixed in 4% paraformaldehyde for 1 week, and embedded in 2% agarose containing 3 mM sodium azide for *ex vivo* MRI (7, 40–42). Imaging was performed on a Bruker 4.7T BioSpec 47/40 Ultra-Shielded Refrigerated nuclear MRI system equipped with a 72 mm I.D. quadrature RF coil and a small-bore (12 cm I.D.) gradient set with a maximum gradient strength of 50 Gauss/cm. MR protocols consisted of a echo-planar diffusion tensor imaging (EP-DTI). Images of 12 contiguous coronal 1 mm slices were obtained with a FOV (field-of-view) of 3.00 cm, a TR of 3,000 ms, TE of 40 ms, and *b*-value of 2,000 mm^2^/s with 30 gradient directions. Brain regions of interest (ROI, Figure 1B) were analyzed by observers blinded to treatment status using Bruker's Paravision 5.1 software. Fractional Anisotropy (FA), Mean Diffusivity (MD, (λ_1_ + λ_2_ + λ_3_)/3), Axial Diffusivity (AD, λ_1_) and Radial Diffusivity (RD, (λ_2_ + λ_3_)/2) were calculated and analyzed.

### Statistical analysis

Normal distribution was verified in all data sets with Shapiro-Wilk test, with Levene's test to confirm homogeneity of variances. For comparison of nonparametric data (performance criteria), Kruskal-Wallis test with Dunn's *post-hoc* test was performed. For analysis of more than two groups with parametric data (sham, CCI-veh and CCI-EPO), two-way ANOVA (injury X treatment) was performed with Bonferroni's *post-hoc* correction for multiple comparisons using SPSS 21 (IBM, Armonk, NY). To test the strength of correlation between the DTI scalars and the primary cognitive outcome, the number of correction trials on reversal testing, Pearson correlations were calculated. The correlations between the P90 MRI and correction trials were performed using the data from each rat. Because the P30 DTI data was from a separate cohort of rats than the adult rats with cognitive data, the P30 imaged brains were randomly assigned within each group to animals undergoing cognitive testing. This process of random assignment followed by correlational analysis was repeated 5 times. Only those ROI that showed significant Pearson correlation on all 5 random assignments AND showed repair of EPO at P30 were considered robust (41). The correlations for a representative random assignment are shown in Table 1. For all analyses, *p* < 0.05 was considered significant.

**Table 1:** Pearson correlation coefficients between diffusion abnormalities repaired by EPO at P30 and poor cognitive flexibility in adulthood.

		**Ipsilateral**	**Contralateral**
**MEAN DIFFUSIVITY**			
Corpus callosum	0.685 *p* < 0.001		
Ventral prefrontal cortex		0.757 *p* < 0.001	0.758 *p* < 0.001
Striatum			0.772 *p* < 0.001
Hippocampus			0.703 *p* < 0.001
Thalamus		0.756 *p* < 0.001	0.783 *p* < 0.001
**AXIAL DIFFUSIVITY**			
Corpus callosum	0.644 *p* = 0.001		
Medial prefrontal cortex		0.660 *p* = 0.001	
Ventral prefrontal cortex		0.707 *p* < 0.001	0.727 *p* < 0.001
Lateral prefrontal cortex		0.567 *p* = 0.006	
Thalamus		0.663 *p* = 0.001	
**RADIAL DIFFUSIVITY**			
Corpus callosum	0.713 *p* < 0.001		
Medial prefrontal cortex			0.693 *p* < 0.001
Ventral prefrontal cortex		0.763 *p* < 0.001	

## Results

### Adult rats subjected to infant TBI can perform VD

We first validated the touchscreen platform in our infant TBI model and assessed whether adult rats subjected to infant CCI could perform VD. Because rats at P10 are approximately equivalent to human infants at term (43, 44), P12 CCI is approximately equivalent to human impact TBI at a few months of age (7). Adult rats in all three groups (sham *n* = 8, CCI-veh *n* = 7, CCI-EPO *n* = 6) successfully completed all aspects of touchscreen habituation and training by P42. Next, we assessed cognitive performance on VD. All rats were able to successfully perform VD. Specifically, 100% of sham and CCI-EPO rats achieved performance criteria, while 83% of CCI-veh rats passed VD. Of those that completed VD, rats across all treatment and injury groups displayed similar numbers of errors and required similar numbers of sessions to pass (Figures 1C,D). Similarly, all rats had comparable reaction time and magazine latency throughout the VD paradigm (Figures 1E,F). Together, these data indicate that rats suffering TBI as pups had the cognitive capacity as adults to complete VD testing.

### Extended EPO treatment prevents deficits in cognitive flexibility induced by infant TBI

**Figure 2:**
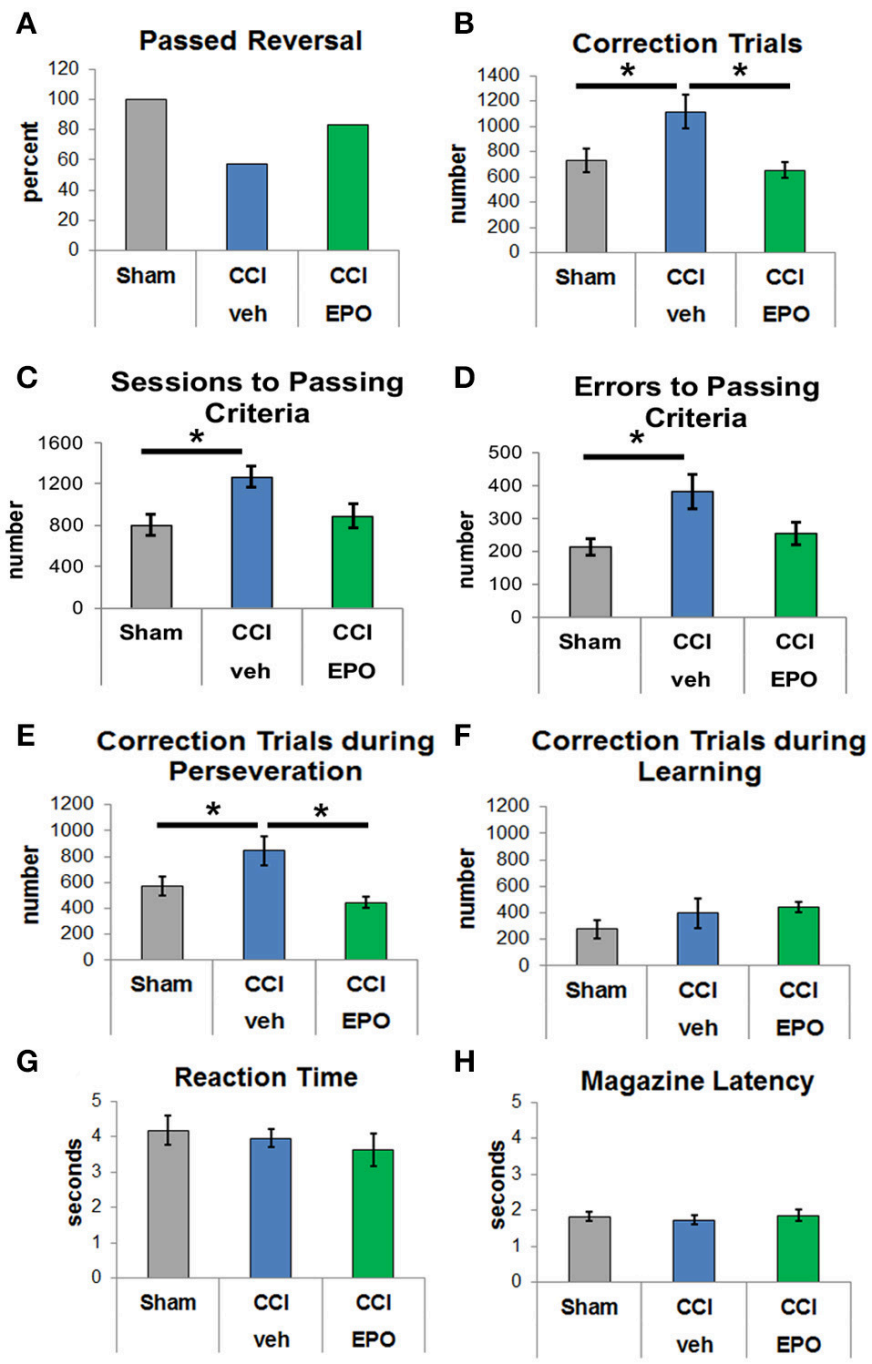
EPO treatment resolves deficits in cognitive flexibility. **(A)** Fewer vehicle-treated CCI rats passed reversal compared to shams or EPO-treated rats. **(B)** Vehicle-treated CCI rats required at least 50% more correction trials than shams or EPO-treated CCI rats (two-way ANOVA with Bonferroni's correction). **(C)** Vehicle-treated CCI rats also required more sessions to achieve passing criteria. **(D)** Similarly, vehicle-treated CCI rats committed more errors prior to reaching passing criteria. **(E)** Importantly, vehicle-treated CCI rats showed significant perseveration and required more correction trials during the perseverative phase than shams or EPO-treated CCI rats. **(F)** By contrast, rats from all 3 groups showed no difference during the learning phase. **(G)** Similar to VD, rats in all 3 groups demonstrated similar reaction times. **(H)** Rats in all three groups exhibited similar motivation to retrieve a reward for a correct response (*n* = 6–8, ^*^p < 0.05).

After successful completion of VD, rats in all groups were evaluated for reversal learning. CCI-veh rats were significantly impaired, and fewer CCI-veh rats passed the reversal-learning paradigm compared to sham and CCI-EPO rats (Figure 2A). Only 57.1% of CCI-veh animals successfully passed criteria compared to 100% of Sham, and 83.3% of CCI-EPO treated animals. Notably, CCI-veh animals required more correction trials (1,114 ± 132) compared to sham (730 ± 95, two-way ANOVA with Bonferroni's correction, *p* = 0.039) and CCI-EPO (652 ± 61, *p* = 0.029, Figure 2B). As expected, CCI-veh animals also required significantly more sessions (Figure 2C) and committed significantly more errors (Figure 2D) to achieve passing criteria. Thus, poor cognitive flexibility in adult rats can be prevented after early TBI by an extended post-injury EPO dosing regimen.

Upon establishing this acquired deficit in cognitive flexibility that was ameliorated with EPO treatment, we analyzed different stages of learning. Specifically, we examined phases of reversal, including the perseverative reversal (<50% correct) and relearning phase (>50% correct). Infant TBI significantly increases maladaptive perseveration during reversal learning (Figure 2E). CCI-veh rats showed high levels of perseveration by responding to the previously rewarded stimulus over several sessions before re-attaining chance. Importantly, the significant increase in correction trials in CCI-veh rats, a measure of perseveration, was prevented with extended post-injury EPO treatment (Figure 2E). During the relearning phase, performance was intact across all three groups with no difference in correction trials (Figure 2F). Changes observed were not due to motivation to respond or retrieve reward, as measured by reaction and reward response times, on either phase of the reversal paradigm (Figures 2G,H). Together, these data emphasize that early TBI affects executive function, specifically cognitive flexibility, and that post-injury EPO treatment results in sustained improvement in cognition.

### Extended EPO treatment yields sustained repair of microstructural brain injury

**Figure 3:**
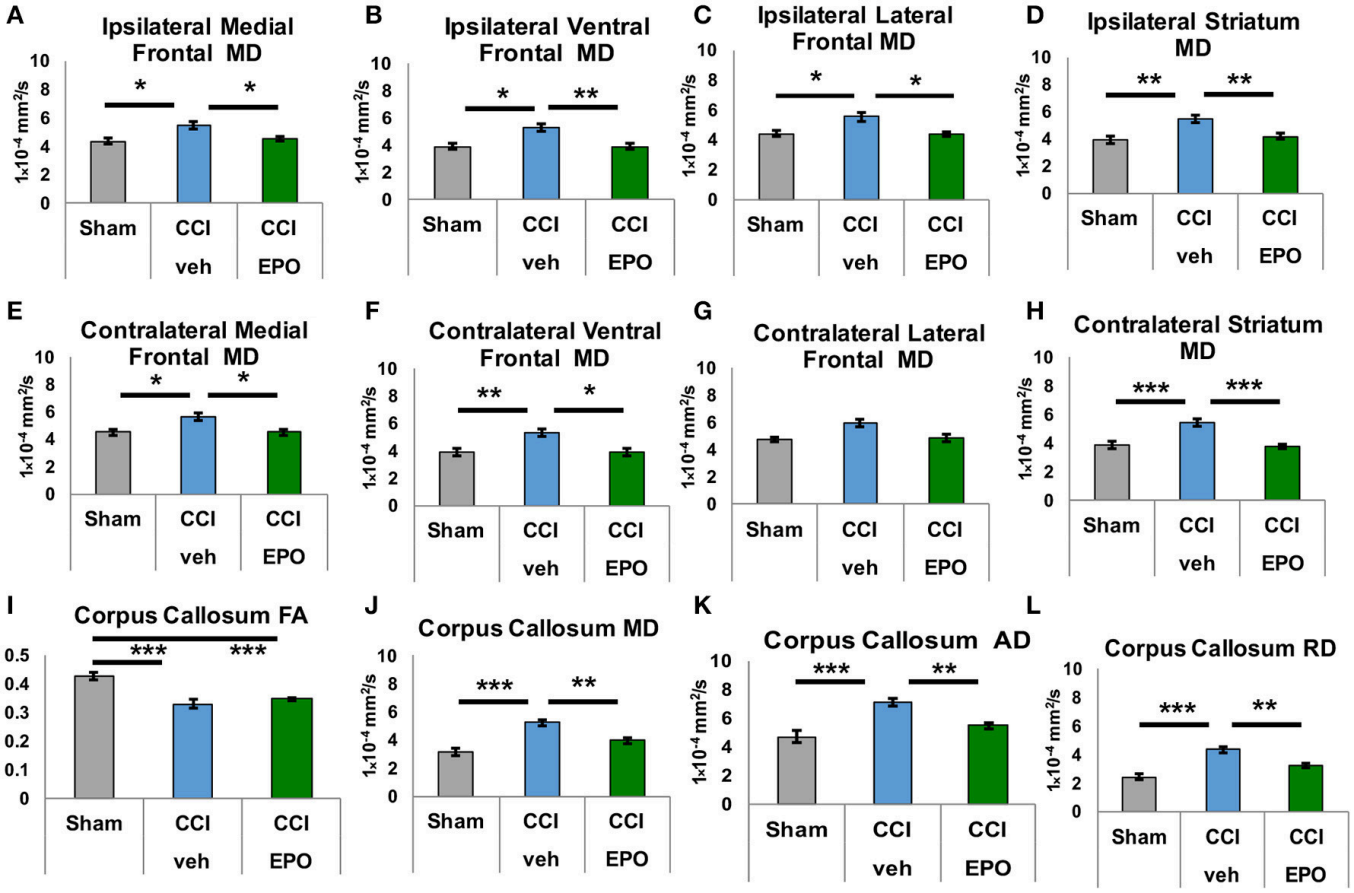
Extended EPO treatment after CCI prevents gray and white matter diffusivity abnormalities in juvenile rats at P30. **(A–C)** Ipsilateral medial, ventral and lateral prefrontal cortices exhibit abnormal mean diffusivity (MD) after CCI that is prevented by EPO treatment. **(E,F)** Similarly, contralateral medial and ventral prefrontal cortices also show abnormal MD that is prevented by EPO treatment. **(G)** Contralateral lateral prefrontal cortex is spared by the injury. **(D,H)** CCI damages deep gray matter striatum MD bilaterally, and EPO treatment prevents theses abnormalities. **(I)** CCI causes loss of FA in white matter corpus callosum that EPO treatment cannot prevent. **(J–L)** CCI induces abnormal mean, axial and radial diffusivity in the corpus callosum that extended EPO treatment prevents (*n* = 6–8, ^*^p < 0.05, ^**^p < 0.01, ^***^p < 0.001).

DTI was performed to more specifically quantify the extent of injury from infant CCI, and the efficacy of EPO treatment on microstructural brain injury. First, we quantified subacute injury in rats following infant CCI at P30, apporoximately 2.5 weeks after P12 infant TBI. Detailed regional analyses of DTI parameters revealed widespread microstructural abnormalities involving the prefrontal cortex, striatum, corpus callosum, hippocampus and thalamus. Similar to prior findings of diffusion abnormalities in bilateral lesional cortex and subcortical white matter after CCI (7), we found widespread reductions in FA in striatum and corpus callosum (Figure 3), and hippocampus and thalamus following injury (CCI-veh *n* = 8), compared to shams (*n* = 6) (Supplemental Figure 3). We also observed robust increases in MD in these regions, including the prefrontal cortex in CCI-veh (Figure 3). Significantly, EPO treatment (*n* = 8) prevented diffusion abnormalities ipsilateral and contralateral to CCI, and normalized MD, AD, and RD in the corpus callosum (Figures 3I–L). EPO treatment also prevented bilateral abnormalities in directional diffusion in the prefrontal cortex, hippocampus and thalamus (Supplemental Figures 1–3). To determine whether subacute alterations in FA and diffusivity predicted later executive function, we tested the correlation between correction trials during reversal learning with DTI metrics at P30. We found robust correlation between cognitive performance and injury following infant TBI in distinct and diverse brain regions essential for cognition including white matter, prefrontal cortex and deep gray matter that are repaired with EPO (Table 1, Supplemental Figure 4).

**Figure 4:**
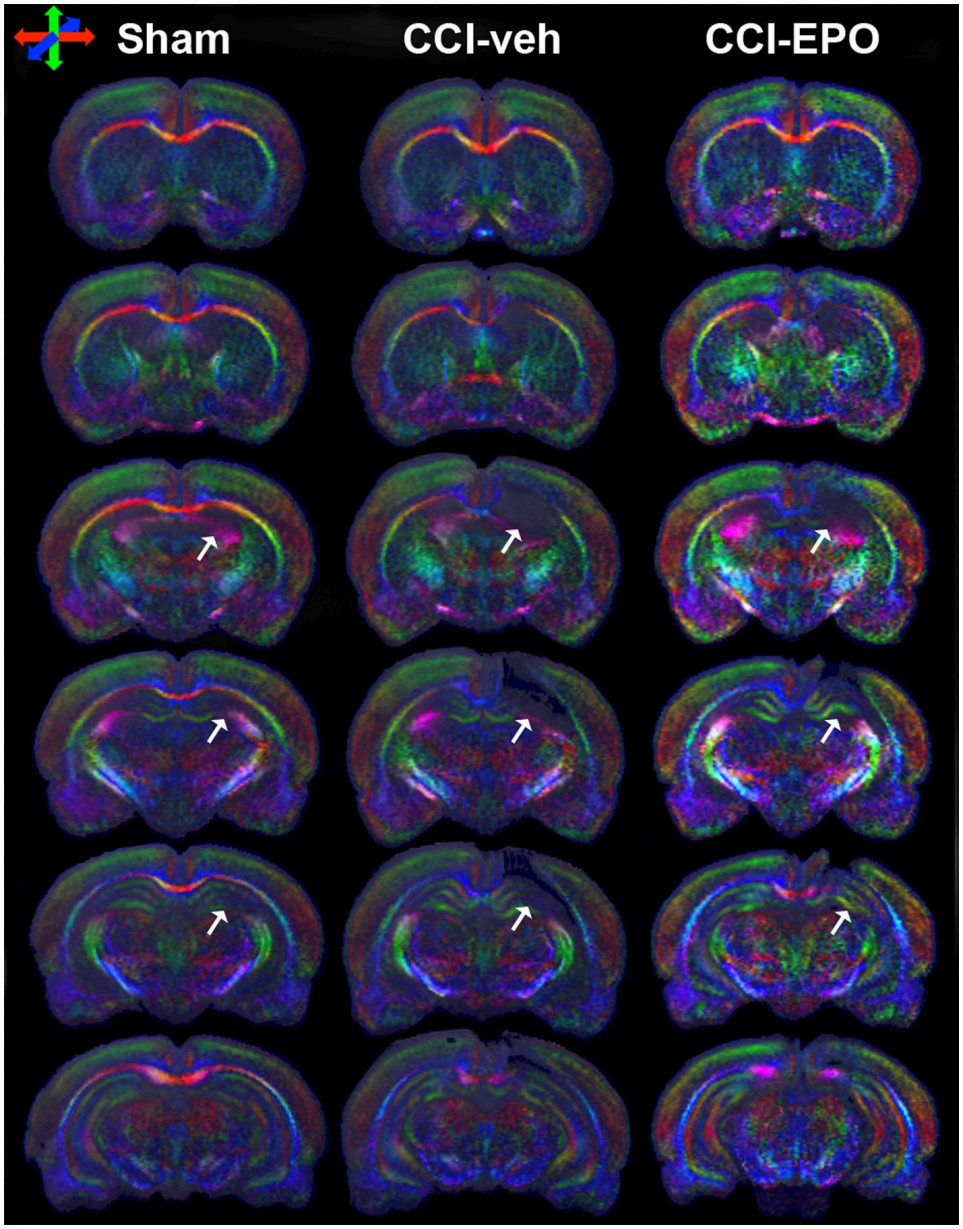
DTI directionally encoded color maps in the coronal plane at P90 demonstrate loss of microstructure in vehicle-treated CCI rats that is not present in EPO-treated CCI rats. White arrows highlight the loss of microstructure and diffusion abnormalities in CCI-veh rats compared to shams, and partial improvement with EPO treatment. Impaired diffusion and loss of microstructure causes loss of directional coherence. (Red–transverse, green–vertical, and blue–orthogonal to the plane).

**Figure 5:**
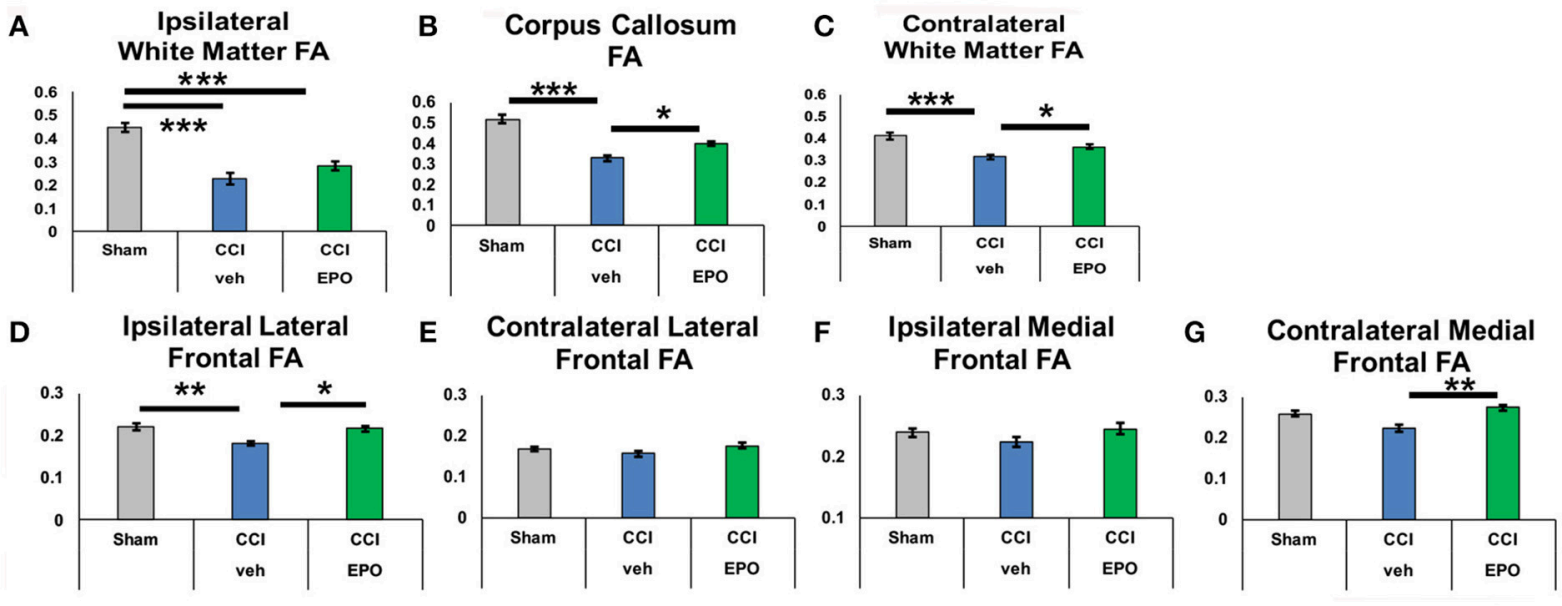
At P90, infant CCI causes widespread chronic damage to the white matter and the ipsilateral lateral prefrontal cortex, and extended EPO treatment prevents sustained injury at a distance from the injury site. **(A)** CCI causes loss of FA in ipsilateral white matter that is not prevented by EPO treatment. **(B,C)** By contrast, white matter loss of FA caused by CCI in the corpus callosum and contralateral white matter is prevented by extended EPO treatment. **(D)** Similarly, EPO treatment after CCI prevents loss of FA in the ipsilateral lateral prefrontal cortex. **(E–G)** CCI does not cause sustained loss of FA in the ipsilateral medial prefrontal cortex, or the contralateral lateral and medial prefrontal cortex (*n* = 6–8, ^*^p < 0.05, ^**^p < 0.01, ^***^p < 0.001).

We next performed DTI on rats at 90 days, immediately following completion of touchscreen assessments (Figures 4, 5). Color maps showed chronic loss of directionality and confirm long-term reductions in structural coherence in cortex and subcortical white matter ipsilateral to CCI, with improvement following EPO treatment (Figure 4). Specifically, FA in corpus callosum and lateral white matter is reduced in CCI-veh animals (*n* = 7) compared to shams (*n* = 7). Significantly, EPO treatment (*n* = 8) prevented abnormal diffusion in the corpus callosum and contralateral subcortical white matter at 90 days (Figure 5B,C). Assessment of the prefrontal cortex confirms loss of FA in the lateral prefrontal regions ipsilateral to impact (Figure 5D), without changes contralateral to impact (Figure 5E). Notably, EPO treatment normalized persistent abnormalities in FA in prefrontal regions after infant TBI (Figures 5D,G). Together, these results suggest that DTI may reflect damage and recovery of the developing brain following early TBI.

To determine if chronic improvement in microstructural integrity in adult (P90) rats indicated improved cognitive flexibility on touchscreen performance, we tested the correlation between correction trials during reversal learning and prevention of FA abnormalities on 90 day DTI. Indeed, extended EPO treatment repair of FA in the contralateral white matter showed significant correlation with executive function (Pearson coefficient −0.532, *p* = 0.013), and the corpus callosum demonstrated a similar, non-significant trend (−0.411, *p* = 0.058). Moreover, the improvement in FA in the ipsilateral lateral prefrontal cortex (−0.471, *p* = 0.042) and medial prefrontal cortex also correlated with improved cognitive performance (−0.557, *p* = 0.011). Thus, resolution of chronic microstructural abnormalities in white and gray matter DTI is related to improved cognitive performance in adult rats following infant TBI, supporting that DTI may be useful as a subacute and chronic biomarker of cognitive outcomes after infant TBI.

## Discussion

In our established model of moderate-severe infantile TBI, extended post-injury EPO treatment prevented widespread bilateral gray and white microstructural injury, concomitant with improved cognitive flexibility. To our knowledge, this is the first demonstration using a translatable, rigorous, touchscreen platform for cognitive testing in a preclinical model of pediatric TBI. Furthermore, EPO, administered in a clinically appropriate extended dosing paradigm compatible with its mechanisms of action, proved efficacious in preventing chronic functional impairment and microstructural brain injury in adult rats following infant TBI. These results emphasize age-appropriate preclinical models with human clinical trial-compatible imaging biomarkers and functional outcome measures. Given the tremendous need for safe, efficacious therapies for neurorepair in the developing CNS after TBI, these data lend support to the testing of EPO in early phase clinical trials for infants with TBI.

Previously, we and others have shown that EPO treatment following perinatal brain injury promotes the genesis, survival, and differentiation of neural cells in the developing and mature CNS, and reduces calpain-mediated injury (7, 19, 21, 35, 40, 41, 45–47). Calpain degrades CNS molecules and proteins essential for the formation of cerebral circuits, including neurofilaments, myelin basic protein and the potassium-chloride transporter, KCC2 (7, 45, 47, 48). Indeed, it is through EPO's signaling action on structural and functional connectivity, neural networks, and excitatory/inhibitory balance of fundamental circuitry that EPO therapy may improve motor and cognitive function following early brain injury, including TBI. Recently, it has been demonstrated that EPO has an additional novel mechanism in regulation of homeostatic plasticity and synaptic strength (17). Together, with previous reports of EPO's modulation of inhibitory circuitry in brain regions key to higher order brain function and structural connectivity (41, 49, 50), and the beneficial impact on neuronal and oligodendroglial differentiation (35, 46, 51), this effect on synapses provides an additional novel molecular mechanism supporting the improvement in cognition and behavior shown here, and the normalization of the trajectory of brain development after perinatal injury. Using this model of infant TBI, we have reported gait deficits, associated serum and inflammatory brain biomarker abnormalities, and a specific subset of MRI changes that are circumvented with extended EPO treatment (7). Here, we significantly expand on these data by demonstrating that infant TBI leads to impairments in executive control that persist into adulthood. These higher-order cognitive processes, which include attention, working memory, future planning and behavioral flexibility, are essential to adult independence and function in an ever-changing environment (37). Notably, development of executive functions depends on prefrontal cortex maturation and integrity, which provide top-down guidance of posterior cortical and subcortical regions (1, 52). Our analyses of pairwise VD learning showed that rats with infant TBI could learn the paradigm comparable to shams. By contrast, adult CCI-veh rats were significantly impaired on reversal learning, consistent with diminished cognitive flexibility. Extended EPO administration after injury normalized adult cognitive performance following TBI and offset perseverative behaviors. Notably, perseverative reversal learning in rodents is mediated by cortical subregions, particularly the lateral prefrontal cortex (37, 53, 54), and DTI following the conclusion of touchscreen testing at 90 days revealed amelioration of abnormal diffusivity in both the lateral prefrontal cortex ipsilateral to impact and in the corpus callosum. The corpus callosum is adjacent to frontal regions, and optimal early reversal in the touchscreen paradigm specifically recruits lateral prefrontal cortex, a region functionally necessary for behavioral flexibility in mice (38, 55). Significantly, microstructural and diffusion injury observed in prefrontal cortex here was attenuated by extended post-injury EPO treatment concomitant with improved structural connectivity in multiple essential networks, emphasizing a putative mechanism for the improvement in executive function observed in our studies.

Touchscreen operant chamber platforms for rodents offer an opportunity to use analogous testing paradigms in humans and preclinical models (34, 56). Touchscreen platforms have been used in humans and rodents to test cognitive deficits related to genetic mutations (38, 39, 57, 58), psychiatric disorders (56, 59) and adult TBI (60, 61). However, to our knowledge, use of touchscreen platforms has not been previously been reported for assessing cognition in pediatric TBI. Similarly, while other investigators have shown EPO optimizes cognitive performance in adult rodents with and without brain injury (22, 35), and examined recognition memory following TBI in the immature brain and subacute recovery with EPO treatment (8, 9), the profile of intact discrimination learning and increased maladaptive perseveration shown here via touchscreen is novel. Indeed, together these data support that early TBI extensively alters CNS development, including frontal cortex, major white matter tracts, and fibers of passage in the parietal and the thalamic relays. Additionally, our data supports a loss of top-down precortical control of striatal subregions (37). The striatum receives input from multiple brain areas including prefrontal cortex, and is fundamental in set-shifting, inhibition and cognitive flexibility (1, 62, 63). We also observed a decrease in bilateral striatal FA at 30 days, together with increased MD. These findings corroborate similar findings in children following TBI who exhibited poor cognition and ventral striatal DTI abnormalities (1). EPO treatment also resolved increases in striatal MD. Taken together, these data indicate repair of microstructural brain injury in major gray and white matter brain regions and strengthen the putative clinical utility of EPO in the context of structural and cognitive recovery following infant TBI. Importantly, clinical findings confirm diminished executive function correlating with decreased structural integrity in the striatum and related structures in adults and children who sustained TBI, providing clinical correlation of our observations (1, 64).

Clinical data confirm that DTI is sensitive to time since injury (65), and an accumulation of evidence implicates resolution of cognitive-behavioral function with altered brain architecture after TBI. DTI measures magnitude and directionality of water diffusion in tissue, and may be a sensitive biomarker of evolving and sustained white matter injury (66). Clinical studies assessing white matter microstructural organization use the same commonly derived diffusion metrics including FA, MD, AD, and RD as investigated here, and confirm children and adolescents with chronic moderate-severe TBI have lower FA and/or higher MD in numerous white matter fiber bundles including the corpus callosum (66–71). Interestingly, MD may index several factors, including fiber density, myelination, and expansion of extracellular space (66, 72). Long-term recovery from TBI is likely dynamic, and the impact of EPO on network function and/or reorganization may be apparent before, or independent from, structural repair. Thus, DTI or similar sophisticated imaging outcomes may serve as a surrogate biomarker to quantify injury and recovery with post-injury interventions.

The strengths of this study include use of high-dose, extended EPO treatment in a clinically relevant dosing regimen after infant TBI. Numerous lines of evidence implicate EPO's utility in the developing brain when administered in a repeated and high-dose regimen and multiple mechanisms of action, including enhanced survival and maturation of oligodendroglial lineage cells (46), reduction in calpain activation (45, 47), decreased inflammation (73), and support of other neural cells, facilitating structural and functional connectivity and contributing to neurorepair in the developing CNS (19, 21, 41). Findings presented here align with neonatal trials using EPO to promote neurorepair (74–76) but are divergent from trials of EPO repair in adult TBI in humans (77, 78) and trials completed in animals without multiple-doses or low dose regimens (79). Notably, EPO repair in the immature brain after injury is distinct from adult TBI trials using EPO due to numerous factors, including developmental mechanisms of action and age-specific pathophysiology related to oligodendroglia, calpain, cell death mechanisms and inhibitory circuit formation, as well as dose, dosing interval, and regimens. Another strength is the use of touchscreen testing to advance the field of cognitive assessment for rodents with early life brain injury. Indeed, a primary challenge in identifying and testing novel interventions to improve cognition is finding paradigms that accurately recapitulate the same function in humans and rodents, and are uncompromised by environmental conditions. An asset of the assessments described here is rigorous control of the performance rules and criteria that distinguish this approach from the use of novel object and water maze assays that use exploration/novelty and stress.

A limitation of the present investigation is that, while both sexes were included throughout, it was underpowered to detect effects of sex in every outcome measure over the developmental time course. Future experiments are warranted with longitudinal, serial multi-modal imaging throughout the acute, subacute and chronic injury periods to fully establish the individual changes in DTI metrics and the correlation with increased cognitive performance. Further studies would also benefit by incorporating advanced examination of networks and connectivity to provide maximum understanding of white matter circuitry following TBI during early and rapid development (1). Future touchscreen investigations in TBI may benefit from the use of a liquid reward if concerns for stress from a mild diet restriction manifest.

In conclusion, Extended EPO treatment restores executive function and prevents microstructural brain abnormalities in adult rats with cognitive deficits in a translational preclinical model of infant TBI. Together with the use of translational touchscreen testing of cognition, these data support the use of EPO in clinical trials for human infants with TBI.

## Author contributions

SR, LJ, RM, and JB conception and design. SR, LJ, LC, JW, AO, TY, JM, YY, and LS acquisition of data. SR, LJ, JB, WM, RM, LS, and NA analysis and interpretation of data. SR and LJ drafting the article. All authors critically revising the article. All authors reviewed submitted version of manuscript. All authors approved the final version of the manuscript. SR and LJ study supervision.

### Conflict of interest statement

The authors declare that the research was conducted in the absence of any commercial or financial relationships that could be construed as a potential conflict of interest. Dr. Meehan receives royalties from ABC-Clio publishing for the sale of his book, *Kids, Sports, and Concussion: A guide for coaches and parents*, and royalties from Wolters Kluwer for working as an author for *UpToDate*. He is under contract with ABC-Clio publishing for a future book entitled, *Concussions*, and with Springer International publishing for a future book entitled, *Head and Neck Injuries in Young Athletes*. His research is funded, in part, by a grant from the National Football League Players Association and by philanthropic support from the National Hockey League Alumni Association through the Corey C. Griffin Pro-Am Tournament.

## Supplementary material

Supplemental Figure 1 Fractional anisotropy (FA) shows minimal regional differences at P30 in prefrontal cortex subregions and striatum. **(A–C,E–G)** No differences in FA are present in prefrontal cortex bilaterally. **(D)** CCI reduces FA in the ipsilateral striatum. **(H)** A similar trend is present in the contralateral striatum (*n* = 6–8,^*^*p* < 0.05).

Supplemental Figure 2 At P30 CCI causes widespread diffusivity abnormalities in prefrontal cortical subregions that are at least partially prevented by extended EPO treatment. **(A–H)** CCI causes bilateral axial and radial diffusivity abnormalities in medial and ventral cortical subregions that are prevented by extended EPO treatment. **(I,K)** While CCI does not cause ipsilateral damage in lateral prefrontal cortex AD, CCI induces contralateral changes in lateral prefrontal cortex AD that are not prevented by EPO treatment. **(J,L)** By contrast, CCI causes alterations in ipsilateral lateral prefrontal cortex RD that are prevented by EPO treatment, while the contralateral lateral prefrontal cortex RD is not affected by CCI (*n* = 6–8, ^*^*p* < 0.05, ^**^*p* < 0.01).

Supplemental Figure 3 At P30 CCI causes diffuse deep gray matter loss of microstructural integrity, and extended EPO treatment prevents these abnormalities at a distance from the injury. **(A,E,I,M)** CCI causes loss of FA in bilateral hippocampal and thalamic subregions, and EPO treatment has minimal impact. **(B–D)** In the ipsilateral hippocampus, CCI causes abnormal mean, axial and radial diffusivity that is also not prevented by EPO treatment, likely due to the proximity to the injury. **(F–H)** By contrast, contralateral abnormalities in hippocampal mean, axial and radial diffusivity are prevented by extended EPO treatment. **(J–L,N–P)** Bilateral thalamic abnormalities in mean, axial and radial diffusivity caused by CCI are also prevented by extended EPO treatment (*n* = 6–8, ^*^*p* < 0.05, ^**^*p* < 0.01, ^***^*p* < 0.001).

Supplemental Figure 4 At P30, abnormal mean diffusivity in vulnerable brain regions that is preventable with EPO treatment (green) correlates with poor cognitive flexibility in adult animals. Area of impact during infancy is shown in pink.
