# Supplementary figures and images for: Extended Erythropoietin Treatment Prevents Chronic Executive Functional and Microstructural Deficits Following Early Severe Traumatic Brain Injury in Rats
Source: Front Neurol. 2018 Jun 19;9:451. doi: 10.3389/fneur.2018.00451 (PMC6018393; doi:10.3389/fneur.2018.00451)

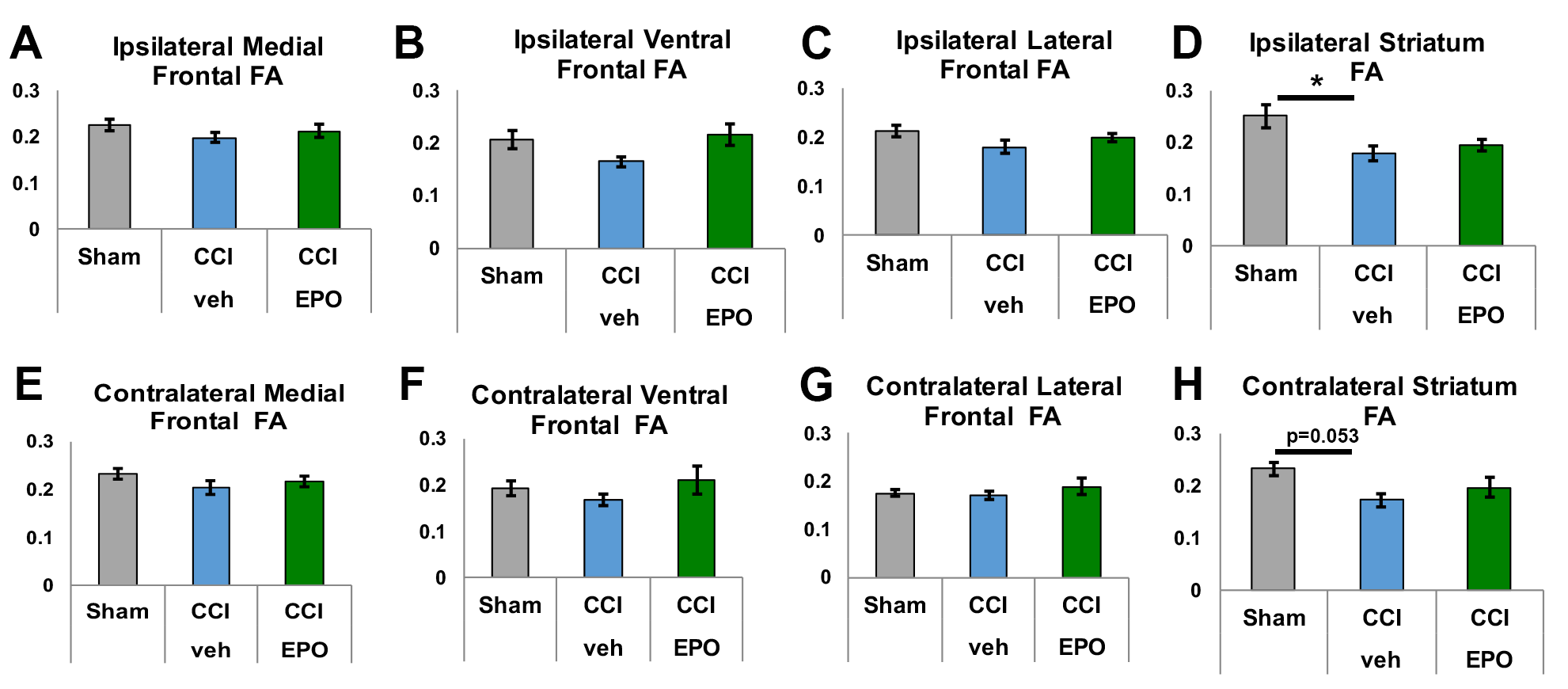

Supplement: Supplemental Figure 1 — Fractional anisotropy (FA) shows minimal regional differences at P30 in prefrontal cortex subregions and striatum. (A–C,E–G) No differences in FA are present in prefrontal cortex bilaterally. (D) CCI reduces FA in the ipsilateral striatum. (H) A similar trend is present in the contralateral striatum (n = 6–8,*p < 0.05). [file Image_1.TIF]

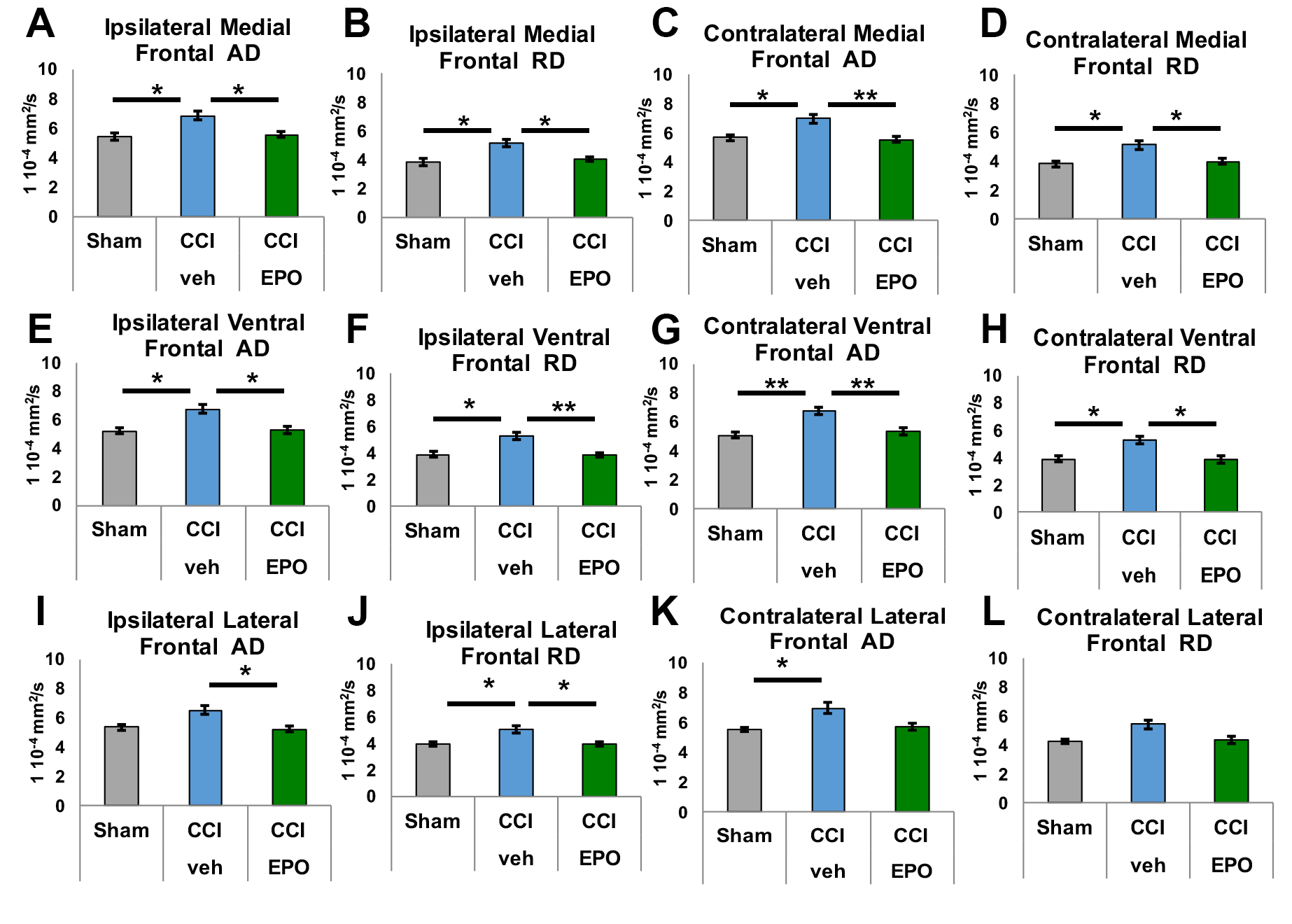

Supplement: Supplemental Figure 2 — At P30 CCI causes widespread diffusivity abnormalities in prefrontal cortical subregions that are at least partially prevented by extended EPO treatment. (A–H) CCI causes bilateral axial and radial diffusivity abnormalities in medial and ventral cortical subregions that are prevented by extended EPO treatment. (I,K) While CCI does not cause ipsilateral damage in lateral prefrontal cortex AD, CCI induces contralateral changes in lateral prefrontal cortex AD that are not prevented by EPO treatment. (J,L) By contrast, CCI causes alterations in ipsilateral lateral prefrontal cortex RD that are prevented by EPO treatment, while the contralateral lateral prefrontal cortex RD is not affected by CCI (n = 6–8, *p < 0.05, **p < 0.01). [file Image_2.TIF]

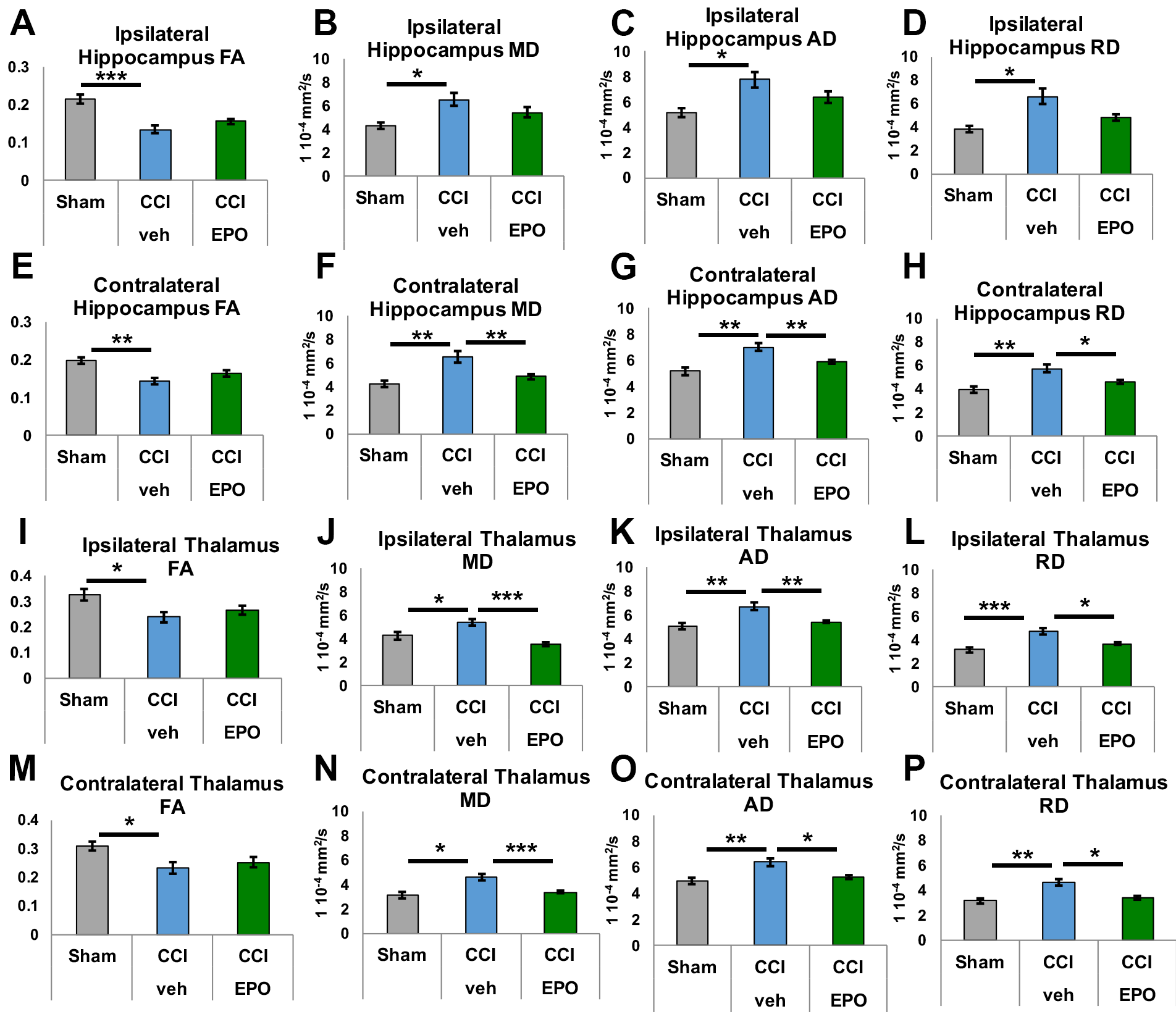

Supplement: Supplemental Figure 3 — At P30 CCI causes diffuse deep gray matter loss of microstructural integrity, and extended EPO treatment prevents these abnormalities at a distance from the injury. (A,E,I,M) CCI causes loss of FA in bilateral hippocampal and thalamic subregions, and EPO treatment has minimal impact. (B–D) In the ipsilateral hippocampus, CCI causes abnormal mean, axial and radial diffusivity that is also not prevented by EPO treatment, likely due to the proximity to the injury. (F–H) By contrast, contralateral abnormalities in hippocampal mean, axial and radial diffusivity are prevented by extended EPO treatment. (J–L,N–P) Bilateral thalamic abnormalities in mean, axial and radial diffusivity caused by CCI are also prevented by extended EPO treatment (n = 6–8, *p < 0.05, **p < 0.01, ***p < 0.001). [file Image_3.TIF]

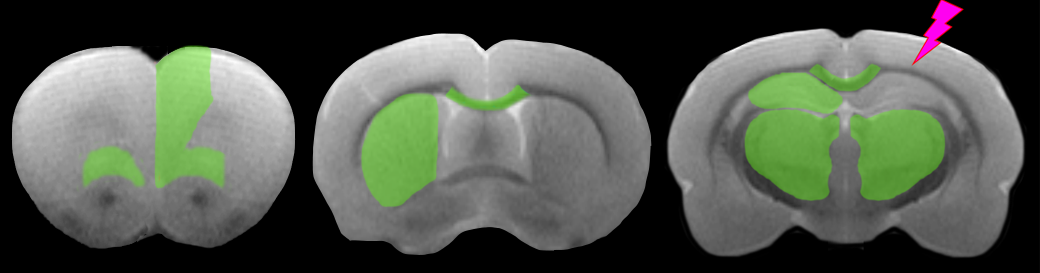

Supplement: Supplemental Figure 4 — At P30, abnormal mean diffusivity in vulnerable brain regions that is preventable with EPO treatment (green) correlates with poor cognitive flexibility in adult animals. Area of impact during infancy is shown in pink. [file Image_4.TIF]
